# Custom-Made Antibiotic Cement-Coated Nail for the Treatment of Infected Bone Defect

**DOI:** 10.1155/2021/6693906

**Published:** 2021-03-05

**Authors:** Guoliang Wang, Wen Luo, Yong Zhou, Zhenfeng Zhu, Zihou Zhao, Shiyu Liu, Jing Li, Xuebin Feng, Yao Zheng, Jiahe Liang, Jiangpu Yi, Yong Zhang, Yunfei Zhang

**Affiliations:** ^1^Department of Orthopaedics, Second Affiliated Hospital, Air Force Medical University, Xi'an, Shaanxi 710038, China; ^2^Department of Ultrasound, Xijing Hospital, Air Force Medical University, Xi'an, Shaanxi 710032, China; ^3^Institute of Oral Tissue Engineering, Air Force Medical University, Xi'an, Shaanxi 710032, China; ^4^Department of Plastic, Second Affiliated Hospital, Air Force Medical University, Xi'an, Shaanxi 710038, China; ^5^Shaanxi Dongwang Technology Co. Ltd., Xi'an, Shaanxi 710075, China; ^6^Department of Pharmacy, Second Affiliated Hospital, Air Force Medical University, Xi'an, Shaanxi 710038, China; ^7^Institute of 3D Printing, Second Affiliated Hospital, Air Force Medical University, Xi'an, Shaanxi 710038, China

## Abstract

**Background:**

Longbone infected bone defect remains a great challenge due to multiple surgeries, long-term treatment duration, and uncertain prognosis. Treatment principles include eradication/debridement, stabilization, and antibiotic administration. An antibiotic cement-coated nail has shown great prospects due to both local antibiotic elution and stabilization of bone defects. However, the current fabrication technique remains to be improved.

**Methods:**

For the first time, we described a new method for custom-made cement-coated nail fabrication based on a 3D printing technique. A retrospective study of 19 consecutive patients with long bone infected bone defects from one medical center was conducted who met the inclusion and exclusion criteria from November 2016 to May 2020. The treatment involved thorough debridement, custom-made antibiotic cement-coated nail filling, and culture-specific systemic antibiotic treatment guided by a multidisciplinary team. Clinical and radiographic examinations (X-ray and CT scans) were used to evaluate bony union. Clinical and laboratory examinations were used to evaluate the infection control. The SF-36 score was used to evaluate patients' quality of life pre- and postoperatively.

**Results:**

The mean follow-up was 98.8 weeks (ranging from 40 to 192). All cases achieved infection control, 3 cases achieved bone healing after one-stage operation, and 12 cases achieved bone healing after a two-stage bone graft procedure. At the last follow-up, none of the 19 patients had infection recurrence or 1 case had failure of the protective plate. The pre- and postoperative SF-36 score showed that there were statistical differences in all the 9 aspects.

**Conclusions:**

The precise custom-made antibiotic cement-coated intramedullary nail through the 3D printing technique used in this study is an effective strategy for the treatment of infected bone defects of long bone. This technique may help to increase the infection control rate and promote bone healing.

## 1. Introduction

Infected bone defect (IBD) is a kind of chronic disease with complex pathology, which usually requires long-term duration and has an uncertain prognosis [1]. Opening fractures, loss of soft tissue or bone tissue, infection after internal fixation, acute or chronic osteomyelitis, and bone tumor are common reasons [2]. Among them, it is more difficult to deal with the infected bone defect after internal fixation, because the internal fixation may help the bacteria to form biofilms which provide a great challenge to the treatment [3–6].

A biofilm is a hydrated matrix composed of polysaccharides and proteins [7]. Once formed, it can protect the pathogenic bacteria from being killed by antibiotics, opsonin, and phagocytosis of white blood cells [8]. To respond to this biofilm-related infection, some researchers suggested the following principles: (1) complete debridement, (2) maintenance of the stability of the defect site, (3) adequate local soft tissue coverage, and (4) effective local and systemic antibiotic concentrations [9, 10]. The traditional treatment of infected bone defects can be described as follows: thorough wound debridement combined with or without local antibiotic, bone cement beads in the medullary cavity, and systemic intravenous antibiotics. The external fixation was usually the first choice to maintain the stability. Through surgical treatment, the infection was firstly controlled to make the infected nonunion become a simple nonunion, and then, the secondary bone graft reconstruction and internal fixation were performed to treat the nonunion [9, 11–15].

However, the traditional treatment has apparent disadvantages: (1) External fixation usually cannot provide enough stability, especially for a large segmental bone defect, which has an adverse effect for infection control or bone union. (2) External fixation has the risk of pain tract infection. (3) The treatment always requires multiple processes of operation. (4) There was a lack of sufficient medullary occupancy effects which may lead to dead cavity formation. (5) To some extent, this method is not conducive to early functional exercise.

Recently, researchers explored intramedullary nails coated with antibiotic cement to treat the infected bone defect, which has apparent advantages compared with the traditional methods. This bone cement-coated nail can reach relatively rigid stability, which is essential to the control of infection and reconstruction of the bone defect. The fully occupied effect of the medulla can avoid the dead cavity which helps to prevent the bacterial colonization. In addition, the bone cement has the antibiotic elution effect which can provide high concentration of local antibiotics higher than the MIC (minimum inhibitory concentration). At the same time, the internal fixation coated with cement can also allow early limb rehabilitation exercise [3, 16]. The use of antibiotic cement-coated internal fixation in the treatment of long bone infected bone defects has been reported in many literatures [3, 6, 16–20].

The current antibiotic bone cement intramedullary nail was usually prepared through an artificial strategy such as manual rolling- [21]), metal mold-, or chest tube mold- [3, 22] based methods ([17–19, 22]; Marcin Krzysztof [23]). However, this artificial wrapping method is usually too complex. In addition, the nail coated with cement usually cannot well adapt to the medullary cavity, which may lead to instability and dead cavity between the nail and medulla. The insertion of the nail is frustrating, and cement damage is common.

In this study, for the first time, we made a custom-made cement-coated nail through an individualized 3D printed mold to achieve better shape adaptation and filling effect. This technique can help to increase the infection control rate and promote bone healing.

## 2. Methods

### 2.1. Participants

19 patients (15 males and 4 females) with the average age of 44.7 (24~67) managed with individual custom-made bone cement intramedullary nails were included in this retrospective study between June 2016 and May 2020. This study was approved by the ethics committee of our institution. All participants provided informed consent preoperatively.

### 2.2. Inclusion Criteria

Patients with focal or segmental infected bone defects of long bone were included in the study. There were no fractures of the same limb or no nerve or main vascular injuries, and the limb should have good soft tissue conditions.

### 2.3. Exclusion Criteria

Patients were excluded who had acute osteomyelitis, severe soft tissue condition, acquired immune deficiency syndrome, diabetes, liver or kidney dysfunction, and systemic malnutrition.

### 2.4. Operative Procedure

Preoperative X-ray, CT, and ECT/CT (sometimes MRI) were routinely performed to evaluate the scope of infection and the area of bone defect. While a team of surgeons performed debridement of the infected defect site, another surgeon prepared the nail on a separate sterile table. We mainly focused on recurrence of infection, bone union, and function as the outcome.

#### 2.4.1. Debridement

**Figure 1:**
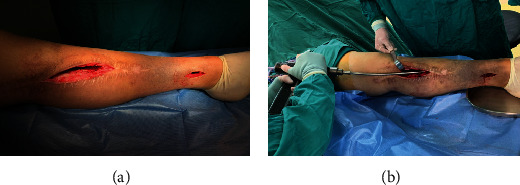
(a) A bone window was made at the distal end of the bone. (b) A soft reaming drill was used to enlarge the medullary cavity to eliminate necrotic and infected tissue.

We paid great attention to the thoroughness of debridement. The previous internal fixations were removed (if there is any), followed by the elimination of the sinus tract and complete debridement of the infected soft tissues. Sometimes, intraoperative frozen-section pathology analysis was performed to clarify the scope of debridement by counting the number of neutrophils under high magnification. The infected bone end was ground with osteotomes until the presence of blood seeping in the bone window (“capsicum sign” positive). The medullary cavity was enlarged with a soft reaming drill, and a bone window was made at the distal end of the bone through which necrosis and infected tissue can be eliminated (Figures 1(a) and 1(b)).

Subsequently, the medullary cavity was irrigated two or three times with sterile saline and hydrogen peroxide. Next, the wound was soaked in dilute iodophor saline for about 5 min, and the surgical drapes and gloves were replaced. Bone and soft tissue samples and purulent secretions were collected for pathology, bacterial culture, and drug sensitivity test.

#### 2.4.2. Preparation of a Custom-Made Cement Nail

**Figure 2:**
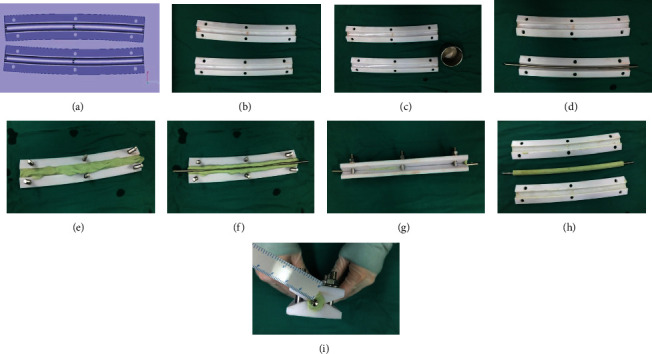
(a) Make the simulated 3D mold, (b) prepare the splint mold through the 3D printing technique, (c) fill the paraffin oil, (d) get a good match, (e) fill the cement to the splint mold, (f) place the nail in the center of the splint mold, and (g) tighten it. (h) The mold was removed after the cement sets. (i) The final diameter of the antibiotic cement-coated nail is 2 mm-6 mm thicker.

*(1) First Step: Preparation of a 3D Printed Mold*. The length, width, and curvature of the medullary cavity were measured by a thin-layer CT scan several days before operation. Then, according to the cavity information, the simulated 3D mold was made (Figure 2(a)). Finally, PETG (polyethylene terephthalate-1,4-cyclohexanedimethanol) was used as the main material to prepare the splint mold through a 3D printing technique (Figure 2(b)). Before the cement coating, the paraffin oil was filled to prevent the adhesion between the mold and cement (Figure 2(c)).

*(2) Second Step: Cement Coating of the Core*. The K wire, commercially intramedullary nail, or individual 3D printed titanium nails were used as the core. The K wire needed to be curved to be well shaped to the canal and mold (Figure 2(d)). 40 g PMMA (polymethylmethacrylate) cement and 2 g antibiotic (vancomycin or imipenem) were mixed; then, the polymer was added. After polymerization, fill the cement to the splint mold (Figure 2(e)), place the intramedullary nail in the center of the splint mold (Figure 2(f)), and then tighten the 2 molds together with 6 screws (Figure 2(g)). The mold was removed after the cement set (usually in 8 to 10 minutes, Figure 2(h)). The final diameter of the antibiotic cement-coated nail is 2 mm-6 mm thicker than the nail itself depending on the size of the core and mold (Figure 2(i)).

**Figure 3:**
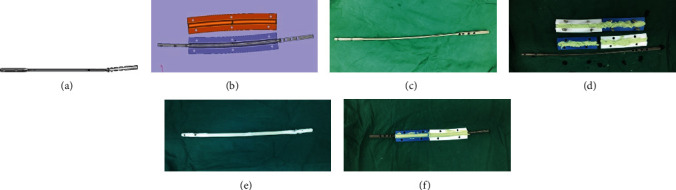
(a, b) The 3D printed custom-made titanium nail and mold were designed. (c, d) The antibiotic cement was coated. (e, f) The finished antibiotic bone cement intramedullary nail.

Except for K wire as the core, a 3D printed individual titanium nail was also tried in this study. After the accurate measurement of the length, width, and curvature of the medullary cavity, the 3D printed custom-made titanium nail and mold were designed (Figures 3(a) and 3(b)). Then, the antibiotic cement was coated as introduced above (Figures 3(c)–3(f)). The diameter of the titanium nail is usually around 5-6 mm which is thinner than the commercially available intramedullary nail for sufficient cement coating.

#### 2.4.3. Insertion of the Nail

The antibiotic cement-coated nail was inserted similarly to any commercial interlocking nail. Usually, reaming of the IM canal was performed to at least 1 mm larger than the diameter of the cement-coated nail. To avoid separation between the cement and nail during insertion, adequate overreaming of the canal was suggested. However, when this does happen, the separated cement has to be removed and a new coated nail will be used.

If the infected bone defect has been converted to defect without infection, exchanging the nail without antibiotic coating and the bone graft can be performed. If there are no infection and union, the antibiotic-coated nail can be left permanently. If both infection and nonunion persist, another antibiotic cement-coated nail should be changed.

### 2.5. Data Collection and Assessment

Patients were required to have follow-up visits in the clinic at 6 weeks and 3, 6, and 12 months after surgery. Baseline data such as age, gender, infection site, etiology, fixation method, and size of defect were collected.

Wound healing and laboratory examinations including white blood cell (WBC) count, C-reactive protein (CRP), and erythrocyte sedimentation rate (ESR) were carried out pre- and postoperatively to analyze the infection control.

X-ray and CT scan were used to assess the quality of union using the Samantha radiographic grading scale to quantify the degree of healing by 2 independent observers [21].

The SF-36 scale was used to assess patients' quality of life from the following eight aspects: physical functioning, role-physical, bodily pain, general health, vitality, social functioning, role-emotional, and mental health. The preoperative and final follow-up results were used to compare and analyze the statistical differences.

### 2.6. Rehabilitation and Antibiotic Regimen

According to the results of drug sensitivity, sensitive antibiotics were used intravenously for about 2 weeks; then, oral antibiotics were used for 4 weeks. After the wound healed, functional exercise of the affected limb joint and gradual partial weight-bearing training under the protection of the brace were allowed. The full weight-bearing was not allowed until the clinical evaluation and imaging evaluation reached bone healing. Imaging examination was performed at 6 weeks, 12 weeks, 6 months, and 1 year after the operation to evaluate the bone healing. For those who need second-stage bone grafting, bone cement can be taken out at a proper time, and the bone grafts can be reconstructed through a membrane-induced technique.

### 2.7. Statistical Analysis

We used Microsoft Excel 2017 software to collect and record data, and IBM SPSS Statistics software (version 23. 0) was used to perform data analysis. Quantitative data were presented as $\bar{x}\pm s$. For preoperative and postoperative SF-36 scores, we conducted the homogeneity test of variance for each group of data, and then, an independent *t*-test was applied. The paired-sample Wilcoxon test was used to analyze WBC, CRP, and ESR levels before and after operation after the orthonormal distribution test. *P* value < 0.05 was considered statistically significant.

## 3. Results

19 patients (14 males and 5 females), with an average age of 44.7, were enrolled in this study. All cases were followed up well, and the longest follow-up time was 48 months. The distribution of infected bone defects was as follows: 1 in the humerus, 7 in the femur, 8 in the tibia, 2 in the radius, and 1 in the clavicle (Table 1).

Etiology analysis was as follows: 8 were opening fractures and 11 were close fractures. 18 were caused by infection after internal fixation. One was caused by infection after external fixation (Table 1).

Infection duration ranges from 3 to 480 months. All 19 patients were analyzed for pathogenic and bacterial culture. 12 of them got positive results. Among them, 4 were methicillin-resistant *Staphylococcus aureus*, 1 was Enterobacter cloacae, 1 was *Staphylococcus epidermidis* mixed with *Enterobacter cloacae*, 1 was *Enterococcus* mixed with *Enterobacter cloacae*, 2 were *Staphylococcus aureus*, 1 was Klebsiella pneumoniae, 1 was hemolytic *Staphylococcus*, and 1 was bizarre. Seven cases were negative in culture but had clinical infection symptoms (Table 1).

### 3.1. Infection Control and Bone Union

According to the results of clinical manifestations and laboratory examination including WBC, CRP, and ESR, after first-stage debridement and custom-made bone cement intramedullary nail operation, all the 19 cases achieved infection control and there was no infection recurrence during the follow-up (Table 2).

Preoperative mean ESR, CRP, and WBC were 13 mm/h, 5.9 mg/L, and 6.45 × 10^9^/L, respectively. The mean ESR, CRP, and WBC 1 week later after operation were 23 mm/h, 6.61 mg/L, and 8.26 × 10^9^/L, respectively, which were all close to the normal range. We conducted statistical analysis on the infection indicators (ESR, CRP, and WBC) before and after the first-stage debridement and custom-made intramedullary nail operation, showing no significant difference between the groups (Table 3).

The area of bone defect ranged from 0.6 cm to 15.0 cm (17.2). Three cases achieved good bone healing after first-stage debridement and custom-made cement intramedullary nail operation, whose defect area was <4 cm. 13 cases achieved bone healing after second-stage internal fixation and bone graft operation. Three cases who were not willing to receive second-stage reconstruction did not achieve bone healing, and they thought that the limb function could meet the basic needs of life after the first-stage operation. One case who did not receive the second-stage reconstruction operation had failure of the protective plate, but the stability provided by the left custom-made intramedullary nail was still good (Table 2).

Therefore, 100% of patients achieved infection control after using the custom-made individual antibiotic bone cement-coated intramedullary nail, which laid the foundation for the second-stage bone grafting operation, and truly achieved the goal of transforming the infectious bone defect into the sterile bone defect.

79% (3 of 19) of the patients achieved direct bone healing only by using custom-made antibiotic cement-coated intramedullary nails.

15.79% (3 of 19) of the patients chose the custom-made antibiotic cement-coated intramedullary nail as the palliative treatment of infectious bone defects.

### 3.2. Life Quality Analysis

We compared the preoperative and postoperative SF-36 scores, and the results showed that there were statistical differences in all 9 aspects (*P* < 0.01), indicating that the postoperative quality of life of patients has been greatly improved (Table 4).

#### 3.2.1. Case 1: Male, 40 y

**Figure 4:**
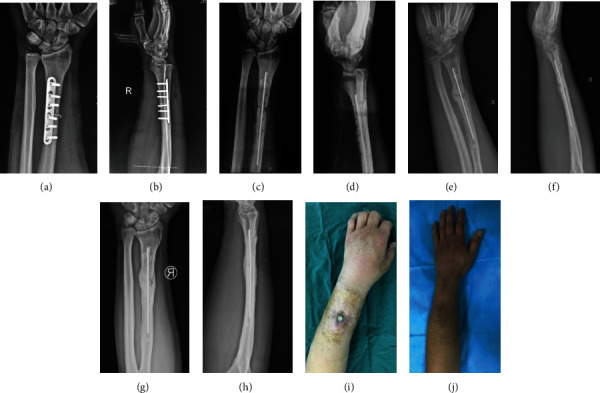
X-ray 3 months after internal fixation of the radial fracture (a, b), after Kirschner wire open placement of bone cement (c, d), at 3 months (e, f), and at 1 year (g, h) postoperatively. General images before (i) and after (j) treatment.

The infection occurred 3 months after plate fixation of the radial shaft fracture. The pathogenic bacteria were MRSA. The plate was removed and debridement was performed, and then, the individualized bone cement intramedullary nail prepared through a 3D printed mold was placed. The infection was well controlled, and the fracture healed after 6 months without additional operation (Figure 4).

#### 3.2.2. Case 2: Female, 30 y

**Figure 5:**
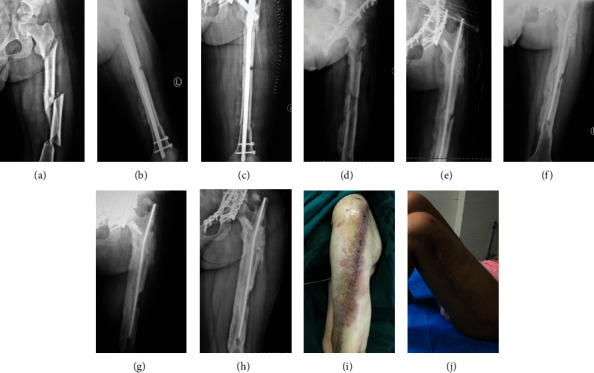
X-ray after the femoral shaft fracture (a), after internal fixation of femoral shaft fractures with the intramedullary nail (b), and at 6 weeks (c) postoperatively. After debridement and removal of internal fixation (d). After Kirschner wire open placement of bone cement (e), at 3 months (f), at 1 year (g), and at 2 years (h). General images before (i) and after (j) treatment.

The infection occurred 3 months after intramedullary nail fixation of the femoral shaft fracture. The pathogenic bacterium was Klebsiella pneumoniae. The initial nail was removed and debridement was performed, and then, the individualized bone cement intramedullary nail prepared through a 3D printed mold was placed. The infection was well controlled, and the fracture healed after 5 months without additional operation (Figure 5).

#### 3.2.3. Case 3: Male, 47 y

**Figure 6:**
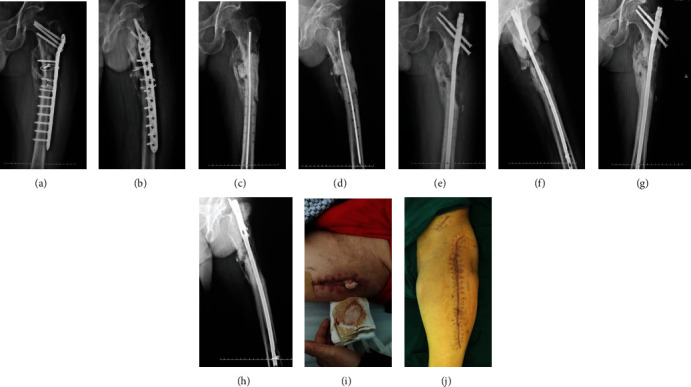
X-ray 1 year after internal fixation of the femoral fracture (a, b), 3 months after Kirschner wire open placement of bone cement (c, d), after the second stage of bone graft internal fixation immediately (e, f), and at 8 months (g, h). General images before (i) and after (j) treatment.

The infection occurred 6 months after plate fixation of the femoral shaft fracture. The pathogenic bacterium was MRSA. The initial plate was removed and debridement was performed, and then, the individualized bone cement intramedullary nail prepared through a 3D printed mold was placed. The infection was well controlled, and the fracture healed after second-state reconstruction surgery (Figure 6).

#### 3.2.4. Case 4: Male, 29 y

**Figure 7:**
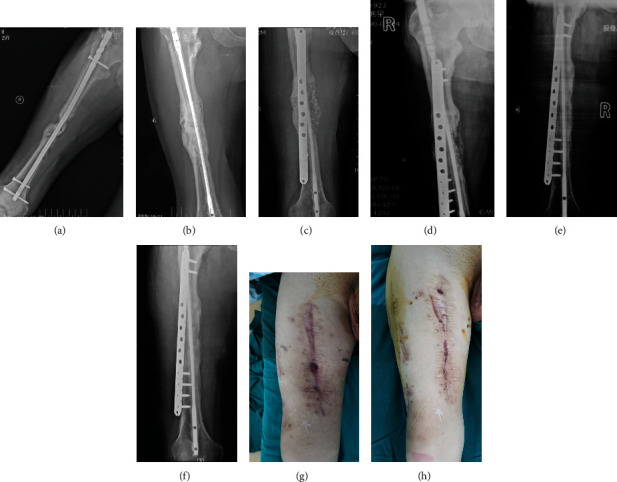
X-ray 1 year after internal fixation of the femoral shaft fracture (a). Debridement of bone cement after intramedullary nailing for 6 months (b). Bone grafting and CS/CP were performed immediately (c), at 6 weeks (d), at 3 months (e), and at 6 months (f). It showed that the CS/CP composite was partially absorbed 3 months after surgery (e), and the rate of absorption was almost comparable to that of new bone formation. Six months after surgery (d), the composite was completely absorbed and the new bone formation was almost complete, and the density of new bone was close to that of the surrounding bone. General images before (g) and after (h) treatment. CS/CP = calcium sulfate and calcium phosphate mixture.

The infection occurred 7 months after intramedullary nail fixation of the femoral shaft fracture. The pathogenic bacterium was MRSA. According to the CT data of the patient's medullary cavity, the individualized 3D printed intramedullary titanium nail was prepared and coated with antibiotic cement by a 3D printed mold. The initial nail was taken out and debridement was performed, and then, the individualized 3D printed antibiotic bone cement intramedullary nail was placed. Infection was well controlled. The second-stage bone grafting and internal fixation were performed, during which the original antibiotic bone cement intramedullary nail was retained with an additional plate augmentation. The bone defect reconstruction was successfully completed 4 months after bone grafting, and the infection did not recur during the follow-up (Figure 7).

## 4. Discussion

Due to the bacterial drug resistance, bacterial biofilm formation, long treatment duration, and uncertain prognosis, infected bone defect has become a great challenge to orthopaedic doctors and patients (Marcin K [24]). There is a lack of universally accepted principles available for the management of infected bone defect. Some researchers suggested the following principles: (1) complete debridement, (2) maintenance of the stability of the fracture end or nonunion site, (3) adequate local soft tissue coverage, and (4) effective local and system antibiotic administration [10, 13].

Conventionally, infected bone defect has been managed through a multiple-stage procedure: firstly to control the infection and subsequently to reconstruct bone defect through internal fixation and bone grafting. Usually, external fixation or antibiotic-impregnated cement beads were used. This traditional procedure has been proven to be effective. However, the disadvantages were also apparent.

Firstly, this traditional strategy requires multiple operation stages. Secondly, the use of external fixation has a relatively high incidence of pin site infections. Sometimes, external fixation with a pin can lead to soft tissue transfixation causing muscle contracture and joint stiffness especially in the case of the femur [25]. Moreover, some obesity patients have poor compliance with this external fixation such as pin site care. Thirdly, to deliver the antibiotic locally to the infection site, the antibiotic-impregnated poly methyl methacrylate cement beads have been always used. However, it cannot provide effect stability for the defect site [26].

The easiest way to reduce infection rates is to time the first internal fixation [27]. For the treatment of open wounds caused by war trauma, early wound disinfection and debridement are very important for the prognosis of open wounds. The best time for wound treatment is 2 hours after the injury. Therefore, early wound treatment can prevent contaminated wounds from becoming infected wounds, which is very critical for the treatment of acute open wounds. We also follow this principle in the treatment of patients with open fractures to achieve early debridement. For open fractures and soft tissue defects due to firearm injuries, the use of cortico-spongioplastics on the basis of a phase I complete debridement of those less than 4 cm obtained favorable results, and the use of the Ilizarov method for those larger than 4 cm had an advantage [28]. Moreover, the Ilizarov method is relatively easy to treat open bone defects caused by firearm injuries, and the operation time is relatively short. For patients with chronic osteomyelitis, infection control is the prerequisite. On the basis of infection control, various methods can be used to repair and reconstruct bone defects. The Ilizarov method is also considered for patients with poor soft tissue conditions who do not use flap repair. For appropriate cases, consider the use of cortico-spongioplastics.

Some researchers try to control the infection and achieve the bone union through a one-step procedure by using antibiotic bone cement-coated internal fixation in the first stage [17].

Through the use of this antibiotic bone cement-coated nail, the issues of anti-infection and stability can be addressed simultaneously. As we know, intramedullary infections always spread along the whole canal, so it will be more effective and adaptable to eliminate the dead space by intramedullary contact [29].

Meanwhile, the local antibiotic eluted from the cement nail may have the bacterial biofilm-killing effect. In addition, the intramedullary nail can provide relatively rigid fixation and stability to the defect site, which will be beneficial to both infection control and bone union [18, 30–32].

The current fabrication method includes manual rolling- or chest tube-based preparation which was arduous and time-consuming [16, 17]. Usually, through this method, the shape of the coated nail cannot well fit the medullary canal cavity, which affects the insertion and the filling effect. Thonse and Conway used an interlocking nail coated with steel molds which can improve the shape of the cement-coated nail [6]. However, the commercial nail has the large diameter which restricts the amount of the cement to only 1-2 mm thick. The thin coating will result in easy damage and limited antibiotic elution effect [32].

To improve the manufacturing process, shorten the fabrication time during the operation, increase the amount of antibiotic bone cement, and ensure the perfect fit between the cement intramedullary nail and the patient's pulp cavity to the greatest extent, we used the individualized 3D printed mold to make the custom-made cement nail.

In most cases, we choose Kirschner wire as the core nail. Of course, the problem is that the mechanical strength is slightly poor, which may not provide adequate stability. However, the bone cement can add strength to the K wire to some extent. Therefore, for some patients, especially for the lower limb, we added bone cement-coated plates to increase the stability.

In order to overcome the shortage of the Kirschner wire, we also made further attempt: the individualized intramedullary titanium nail with a thinner diameter compared with a commercial nail was prepared through 3D printing, and then, antibiotic cement was coated by using a custom-made 3D printed mold. This method has apparent advantages: it has increased mechanical strength compared with Kirschner wire, has increased the amount of antibiotic bone cement by reducing the diameter of the intramedullary nail, and has better shape fit in the canal.

Some authors think that the effect of a prefabricated good bone cement liner is better than that of handmade cushion [33]; We agree with this view, because preformed stents allow for better personalized treatment, better stent-wound match, better stability, more effective placeholders, and more effective sustained-release antibiotics. A prefabricated 3D printed intramedullary nail was also used to treat bone infection, but due to the complexity of trauma patients, the diversity of lesion sites, and the ease of implantation, the bone cement coating on the surface of the intramedullary nail was improvised intraoperatively. Another reason is that there is no prefabricated finished placeholder designed for trauma patients. If there is a suitable product in the later stage, we will try to use it in the clinical work.

In this study, we used vancomycin or meropenem as an antibiotic loaded on bone cement. Osteomyelitis is usually caused by a variety of microorganisms, and the most common pathogenic bacteria reported in the literature are *Staphylococcus aureus* [34]. *Staphylococcus aureus* is accompanied by other pathogenic bacteria in 65%-70% of cases. Gentamicin, tobramycin, meropenem, and vancomycin are common local antibiotics because of their broad antibacterial spectrum, good thermal stability, and low sensitivity [35]. In previous studies, vancomycin and gentamicin or tobramycin were selected more frequently [3, 18, 22]. In this study, 7 in 19 cases were Staphylococcus, of which all were sensitive to vancomycin, 5 in 19 were Gram-negative bacilli, of which they were sensitive to meropenem, 1 was *Enterobacter cloacae*, 1 was *Klebsiella pneumoniae*, 1 was *Proteus mirabilis*, 1 was *Staphylococcus* mixed with *Enterobacter cloacae*, 1 was *Enterococcus* mixed with *Enterobacter cloacae*, and other was negative. The local antibiotic was selected according to the results of bacterial culture and drug sensitivity in most cases. Increasing drug sensitivity results show that deep pathogenic bacteria are resistant to gentamicin, including intramedullary infection [11].

As to the systemic antibiotic use strategy, pharmacology experts were specially invited to guide the use of systemic antibiotics. Cefotiam, which can cover both Gram-negative and Gram-positive bacteria, was routinely used 24 to 48 hours after surgery, before bacterial culture and drug sensitivity results were obtained. After the drug sensitivity results come out, the sensitive antibiotic will be used until 4-6 weeks including intravenous and oral medication.

Currently, the total course of treatment for bone infections is generally considered to be more than six weeks, but there is some debate over whether to give it intravenously or orally. Some authors have reported that oral antibiotics are as effective as intravenous antibiotics in the first 6 weeks of infection. At the same time, oral antibiotics can reduce the length of hospital stay and avoid the occurrence of cross-infection during hospital stay (H.-K. [36]). As for the specific duration of intravenous antibiotics, we should also refer to the results of bacterial culture and the suggestions of clinical pharmacists. According to our experience, the duration of intravenous antibiotics is generally 2-3 weeks. In addition, oral antibiotics of 4 weeks should be combined to ensure that the duration of antibiotic treatment is not less than 6 weeks.

In this study, all cases of infection were controlled in one stage, indicating the advantages of the individualized intramedullary nail in the infection control effect.

Paley and Herzenberg reported infection control in the 9 cases [18]. Thonse and Conway reported an 85% rate of infection control [6]. Bhatia et al. reported a 95% rate of infection eradication [37]. Similar results proved that the bone cement nail has a satisfactory infection control effect [38].

In terms of bone union, there are different results in different studies. Bhatia et al. reported a 60% rate of bone union without an additional procedure [37]. Zheng and Hang reported a 22% rate of union [19]. Thonse and Conway reported a 73% union rate [6]. In this study, 3 cases healed directly in the first stage whose bone defect range was smaller than 4 cm which is similar to Shyam et al.'s report [22, 37, 39]. However, due to the small number of cases, it is hard to analyze the correlation between the extent of bone defect and the primary healing effect. Some patients did not receive the second stage of bone grafting and did not have bone healing. The reason is that they thought that the limb function and life quality after the first individual intramedullary nail operation were satisfactory, but these patients were told to have crutch protection.

Recent studies suggest that the use of teriparatide combined with the Ilizarov method in the treatment of blood-borne osteomyelitis can accelerate the mineralization rate of new bone after bone removal, thus enabling the removal of the external frame as soon as possible [40]. Teriparatide is a drug to promote bone formation. It is a good way to accelerate bone healing by combining drugs with surgery, so as to improve the success rate of surgery and obtain good results. In clinical practice, if we meet similar appropriate cases, we can also try. However, most of the patients receiving treatment at present have poor economic conditions, and the use of teriparatide treatment virtually increases the economic burden of patients, which is also a problem to be considered in the treatment.

Currently, PMMA is routinely used for the coating of nails. However, PMMA is nondegradable and can be difficult to remove surgically. In addition, thermal reaction, antibiotic release profile, and toxicity effects were still the concern. Anugraha et al. used a calcium sulfate hydroxyapatite biocomposite to coat the nail, which may provide new alternatives [34].

There are certain shortcomings in our study. This was a retrospective clinical study with a relatively small number of cases. The infection site of bone is scattered and not concentrated. The mineral oil on the surface of the nail may lead to local inflammatory responses. The biomechanics of this bone cement intramedullary nail require further investigation.

## 5. Conclusions

The custom-made 3D printed antibiotic cement-coated intramedullary nail used in this study has better filling effect and apparent advantages for the treatment of infected bone defect of long bone. The rate of infection control is high, and some cases can achieve direct bone healing. Compared with the bone cement chain bead, it has good occupying effect and can provide stability. Compared with the external fixation, it does not need complex external fixation frame assembly and has good patient compliance.

## Figures and Tables

**Table 1:** 

Patient no.	Age/sex	Bone	Classification	Initial treatment	Duration of infection (month)	Organism
1	49/M	Radius	Closed	LCP	3	MRSA
2	40/F	Radius	Closed	LCP	3	MRSA
3	30/F	Femur	Closed	ILN	3	Klebsiella
4	34/M	Tibia	Closed	LCP	9	—
5	45/M	Clavicle	Closed	LCP	8	—
6	44/M	Tibia	IIIB	Deb, ex-fix	3	*Enterobacter cloacae*+*Enterococcus raffinosus*
7	54/F	Tibia	IIIB	Deb, LCP	9	—
8	24/M	Tibia	Closed	LCP	3	*Enterobacter cloacae*
9	67/M	Tibia	IIIB	Deb, LCP	417	*Staphylococcus haemolyticus*
10	33/M	Femur	II	Deb, ILN	8	—
11	46/M	Tibia	IIIC	Deb, LCP	4	*S. aureus*
12	29/M	Tibia	IIIA	Deb, LCP	108	—
13	29/M	Femur	II	Deb, ILN	7	MRSA
14	60/M	Femur	IIIA	Deb, ILN	12	*S. aureus*
15	42/M	Femur	Closed	ILN	4	*Enterobacter cloacae*+MRSE
16	47/M	Femur	Closed	LCP	5	MRSA
17	59/F	Humerus	Closed	LCP	3	—
18	53/M	Tibia	Closed	LCP	3	—
19	64/M	Femur	Closed	LCP	480	Proteus mirabilis

Classification according to the Gustilo Anderson classification. Deb = debridement; ILN = intramedullary interlock nailing; ex-fix = external fixator; LCP = locking compression plate.

**Table 2:** 

Patient no.	Bone defect (cm)	Infection control	Duration of infection control (weeks)	Secondary procedure	Healing condition
Group1 (bone defect < 4 cm)					
1	0.6	Control	12	None	Primary healing
2	1.5	Control	16	None	Primary healing
3	2.0	Control	15	None	Primary healing
4	2.1	Control	24	None	—
5	2.5	Control	12	BG+LCP	Healing
Group 2 (bone defect ≥ 4 cm)					
6	4.4	Control	26	CS/CP+BG+LCP+ex-fix	Healing
7	5.3	Control	13	None	—
8	5.6	Control	16	CS+BG	Healing
9	6.0	Control	12	None	—
10	6.3	Control	12	BG+LCP+ILN	Healing
11	7.5	Control	16	CS/CP+BG+ILN	Healing
12	9.4	Control	13	CS/CP+BG	Healing
13	9.4	Control	16	CS/CP+BG+LCP	Healing
14	9.8	Control	12	BG+LCP+ILN	Healing
15	10.4	Control	12	BG+LCP	Healing
16	11.6	Control	12	CS+BG+ILN	Healing
17	12.9	Control	32	CS/CP+BG+LCP	Healing
18	12.9	Control	16	CS+BG+ILN	Healing
19	15.0	Control	12	None	—

Deb = debridement; ILN = intramedullary interlock nailing; ex-fix = external fixator; LCP = locking compression plate; BG = bone graft; CS = calcium sulfate; CS/CP = calcium sulfate and calcium phosphate mixture.

**Table 3:** Preoperative and postoperative infection-related indicators.

Item	Before	After	*P*
WBC (/L)	6.45 (5.4, 8.91)	8.26 (5.41, 10.59)	0.546
ESR (mm/h)	13 (7, 46)	23 (10, 34)	0.758
CRP (mg/L)	5.9 (2.01, 27.75)	6.61 (2.78, 15.63)	0.609

**Table 4:** Preoperative and postoperative SF-36 scale scores $\left(\bar{x}\pm s\right)$.

Classification	Preop SF-36 score	Postop SF-36 score	*P*
Body pain	40.26 ± 20.63	93.03 ± 12.92	<0.001
Physical functioning	37.68 ± 26.21	69.21 ± 23.94	<0.001
Role-physical	3.95 ± 12.54	61.84 ± 39.41	0.001
General health	33.95 ± 22.58	58.95 ± 12.20	<0.001
Vitality	42.11 ± 22.69	75.00 ± 12.02	<0.001
Social functioning	27.63 ± 21.88	76.32 ± 21.20	<0.001
Role-emotional	26.32 ± 39.41	77.19 ± 38.57	0.001
Mental health	44.84 ± 19.58	69.26 ± 13.47	<0.001
Reported health transition	26.32 ± 28.23	92.11 ± 14.56	<0.001

## Data Availability

The data used to support the findings of this study were supplied by YUNFEI ZHANG under license and so cannot be made freely available. Requests for access to these data should be made to tdbone@163.com.

## Abbreviations

IBD: Infected bone defect
MIC: Minimum inhibitory concentration
PETG: Polyethylene terephthalate-1,4-cyclohexanedimethanol.
